# Editorial: Non-Dopaminergic Systems in Parkinson’s Disease

**DOI:** 10.3389/fphar.2020.593822

**Published:** 2020-09-11

**Authors:** Cristina Miguelez, Philippe De Deurwaerdère, Véronique Sgambato

**Affiliations:** ^1^ Department of Pharmacology, Faculty of Medicine and Nursing, University of the Basque Country (UPV/EHU), Leioa, Spain; ^2^ Autonomic and Movement Disorders Unit, Neurodegenerative Diseases, Biocruces Health Research Institute, Barakaldo, Spain; ^3^ CNRS, Institut des Neurosciences Cognitives et Integratives d’Aquitaine, UMR 5287, Bordeaux, France; ^4^ University of Lyon, CNRS, Institut des Sciences Cognitives Marc Jeannerod, UMR 5229, Bron, France

**Keywords:** L-DOPA, serotonin, sleep, noradrenaline, GABA, non-motor symptoms, orphan receptor, prodromal Parkinson disease

This collection presents an array of articles addressing the non-dopaminergic mechanisms in Parkinson’s disease (PD). The dopaminergic system has been the main focus for almost 70 years and dopaminergic-based strategies still remain the best symptomatic medication to improve quality of life of parkinsonian patients. However, the interest in non-dopaminergic systems, even beyond neurotransmission itself, has grown with the accumulation of data showing that PD cannot be considered as a pure motor disease and understood only through the prism of the nigrostriatal dopaminergic pathway deficiency. Indeed, the disease progresses through different stages and damages to other areas and neurochemical systems precede the degeneration of dopaminergic neurons in the substantia nigra compacta (SNc). The other affected systems participate in the so-called prodromal or pre-symptomatic phases and contribute to the malfunctioning of the motor and non-motor circuits. Meanwhile, the non-motor symptoms are increasingly recognized in PD. They severely alter the quality of life of the patients since they are often more debilitating than the motor ones and poorly respond to dopaminergic-based therapies. Finally, the studies addressing the mechanisms of action of antiparkinsonian drugs have revealed that their benefits/side effects involved mechanisms other than the dopaminergic ones.

Among the classical non-dopaminergic systems involved in PD, Paredes-Rodriguez et al. review the situation of the noradrenergic system in PD. The noradrenergic system issued from the locus coeruleus can display severe damages in the disease, presumably before the dopaminergic neurons according to the hypothesis that the disease progresses from the caudal to the rostral parts of the brainstem. In fact, the authors recall that the noradrenergic system exerts anti-inflammatory and neuroprotective effect on the dopaminergic degeneration, and noradrenergic damage can consequently favor the progress of the disease. Even though the noradrenergic system participates in the mechanism of action of L-DOPA, this interaction is still unclear and needs further investigation. Mallet et al. review another classical non-dopaminergic neurotransmitter that has been considered in PD even before dopamine, namely acetylcholine. The authors focus their analysis on the striatal cholinergic interneurons, which display specific electrophysiological features but are still difficult to apprehend due to their sparse distribution. Based on recent literature, the authors report the advantages of using optogenetic approaches possibly combined with computational studies to investigate the role of cholinergic interneurons in the physiology and pathophysiology of motor behavior and cognition. On the other hand, the serotonergic system is one of the most studied non-dopaminergic neurotransmitter systems during the last decade in view of the demonstration of its primary participation in dyskinesia induced by L-DOPA. Schintu et al. rendered hemiparkinsonian inbred depression-like flinders sensitive line (FSL) rats and studied their motor responses to chronic L-DOPA. In contrast to control animals, the hemiparkinsonian FSL rats did not display sensitization in the turning response to apomorphine or L-DOPA and only a weak increase in dyskinesia. The marked differences in L-DOPA-induced dyskinesia were not associated with modification of the striatal expression of deltaFosB, a supposed marker of this side effect.

The imbalance of neurotransmitters in the brain is multiple in part due to degenerative processes away from the SNc. Chambers et al. recall that some neurons in the pedunculopontine nucleus, a complex structure housing GABAergic, glutamatergic, and cholinergic neurons, degenerate in PD. The neuronal degenerative process in the pedunculopontine nucleus likely participates in motor disability, notably gait control, and in non-motor symptoms including sleep deficits, likely due to the innervation of brain regions different from the SNc. Murueta-Goyena et al. cover the non-motor symptoms in PD and the GABAergic system alterations. They recall the participation of GABAergic mechanisms in various pre-symptomatic disturbances including olfactory loss, gastrointestinal abnormalities, visual alterations, cognitive and mood disorders, and make a special emphasis on rapid eye movement sleep behavior disorder. In this line, Meideiros et al. describe how neurotoxic and genetic manipulations of rodents have been utilized to reproduce some of the major sleep disturbances associated with PD. They further discuss how these abnormalities can be linked to noradrenaline, serotonin, and orexin transmission. Several neurotransmitter imbalances operate during the disease and may participate in the complexity and heterogeneity of the neuropsychiatric symptoms exhibited by PD patients. Dujardin and Sgambato propose a thorough examination of the neuropsychiatric symptoms, including depression, anxiety, apathy, psychosis, and impulse control disorders, their prevalence and pathophysiology. The authors deepened their analysis by looking at the relative contribution of the neurotransmitter systems dopamine, noradrenaline, serotonin, acetylcholine, GABA, and glutamate in each of the above-mentioned neuropsychiatric symptoms in both PD patients and animal models.

Beyond the neurotransmitter systems, there are some opportunities to develop treatments on specific targets, as the orphan receptors GPR88 and GPR143. Ingallinesi et al. focused on GPR88, almost exclusively expressed by the medium spiny GABAergic neurons of the striatum. They used a lentiviral-mediated knock-down approach of the receptor in the 6-hydroxydopamine neurotoxic rat model of PD and reported reduced amphetamine- but increased L-DOPA-induced turning behavior. These behavioral effects were paralleled by a normalization of some striatal tissue markers such as GAD67 expression, although the striatal expression of deltaFosB did not parallel L-DOPA-induced dyskinesia. Yoshio et al. also recall the interest of another orphan receptor, the GPR143. This receptor has been proposed as the target of L-DOPA, which would be its endogenous ligand at least in the retina. The authors review the possible contribution of GPR143 in PD with a special emphasis on its colocalization with the protein *α*-synuclein in Lewy bodies.

The availability of new animal models conditions the progress of research in the involvement of different neurotransmitters in the motor and non-motor symptoms of the disease. Gomez-Benito et al. discuss and provide detailed comparative analysis of two models based on *α*-synuclein: the *α*-synuclein pre-formed fibril model and the recombinant adeno-associated virus vector-mediated *α-*Synuclein overexpression models. The multiplicity and development of novel models are necessary for progressing in the understanding of the disease and help to decipher specific mechanisms. In any case, far beyond the consideration of dopaminergic neurons of the SNc, PD is a multifactorial disease evolving before the onset of motor symptoms.

## Author Contributions

All authors contributed to the article and approved the submitted version.

## Conflict of Interest

The authors declare that the research was conducted in the absence of any commercial or financial relationships that could be construed as a potential conflict of interest.

